# Thermal Stability of P-Type BiSbTe Alloys Prepared by Melt Spinning and Rapid Sintering

**DOI:** 10.3390/ma10060617

**Published:** 2017-06-06

**Authors:** Yun Zheng, Gangjian Tan, Yubo Luo, Xianli Su, Yonggao Yan, Xinfeng Tang

**Affiliations:** 1 State Key Laboratory of Advanced Technology for Materials Synthesis and Processing, Wuhan University of Technology, Wuhan 430070, China; zheng.yun@ntu.edu.sg (Y.Z.); suxianli@whut.edu.cn (X.S.); yanyonggao@whut.edu.cn (Y.Y.); 2 School of Materials Science and Engineering, Nanyang Technological Unviersity, Singapore 639798, Singapore; luoyubo@ntu.edu.sg; 3 Department of Chemistry, Northwestern University, Evanston, IL 60208, USA; gangjian.tan@northwestern.edu

**Keywords:** thermoelectric, thermal stability, BiSbTe alloys, melt spinning

## Abstract

P-type BiSbTe alloys have been widely implemented in waste heat recovery from low-grade heat sources below 600 K, which may involve assorted environments and conditions, such as long-term service, high-temperature exposure (generally 473–573 K) and mechanical forces. It is important to evaluate the service performance of these materials in order to prevent possible failures in advance and extend the life cycle. In this study, p-type Bi_0.5_Sb_1.5_Te_3_ commercial zone-melting (ZM) ingots were processed by melt spinning and subsequent plasma-activated sintering (MS-PAS), and were then subjected to vacuum-annealing at 473 and 573 K, respectively, for one week. The results show that MS-PAS samples exhibit excellent thermal stability when annealed at 473 K. However, thermal annealing at 573 K for MS-PAS specimens leads to the distinct sublimation of the element Te, which degrades the hole concentration remarkably and results in inferior thermoelectric performance. Furthermore, MS-PAS samples annealed at 473 K demonstrate a slight enhancement in flexural and compressive strengths, probably due to the reduction of residual stress induced during the sintering process. The current work guides the reliable application of p-type Bi_0.5_Sb_1.5_Te_3_ compounds prepared by the MS-PAS technique.

## 1. Introduction

Thermoelectric (TE) energy generation has emerged as a potential technology for energy harvesting from low-grade heat sources—such as solar sources, geothermal heat, and waste heat from vehicles, vessels and power plants—in order to alleviate the energy crisis and promote the sustainable development of our society [1]. TE power generation is based on TE materials, which can realize the direct conversion of heat into electricity. The performance of TE materials is evaluated by the dimensionless figure of merit *ZT* = *α*^2^*σT*/*K*, where *α*, *σ*, *K*, and *T* refer respectively to the Seebeck coefficient, electrical conductivity, thermal conductivity and absolute temperature [2,3]. The efficiency of TE energy conversion depends on the operation temperature and figure of merit *ZT*, and can be increased by maximizing the average *ZT* value [4,5,6]. The recent decades have witnessed explosive developments in diversified materials with greatly enhanced TE performance, such as IV-VI alloys (typically SnSe [7,8,9], PbTe [10,11,12], GeTe [13,14], etc.), which have exceeded the value of *ZT* = 2.0. However, only Bi_2_Te_3_-based alloys have been proven to be the most mature TE materials, and have been commercialized for industrial application for decades [15]. The demands are continuously growing for Bi_2_Te_3_-based modules with a widely-expanding market in power generation from low-grade waste heat (generally <600 K) [16,17,18,19]. The zone-melting (ZM) method has been employed in the mass production of commercial Bi_2_Te_3_-based ingots with considerable TE performance (generally around unity). However, due to the easy cleavage nature along basal planes, ZM ingots demonstrate poor mechanical properties and machinability, which has hampered the manufacturing of TE modules with micron-sized legs and long-term reliability [20,21,22]. In recent years, great breakthroughs have been made in achieving high TE performance and mechanically robust Bi_2_Te_3_-based materials by numerous methods, such as nanostructuring [23,24,25], hybriding [26,27,28,29,30,31] and defects engineering [32,33,34,35]. Among these advanced technologies, melt spinning combined with plasma activated sintering (MS-PAS) has been proven to be an effective way of introducing hierarchical structures to significantly reduce the lattice thermal conductivity without degrading the electrical conductivity [21,36]. Additionally, MS-PAS can result in enhanced mechanical properties, showing its great potential in practical applications.

It is noteworthy that commercialized Bi_2_Te_3_-based materials will inevitably suffer from some of the following harsh environments while in service: (1) long-term operation (tens of thousands of hours); (2) high temperature exposure (473–573 K); or (3) various stresses arising from mechanical vibration, clamping, or mismatching of thermal expansion coefficients between the electrodes, barrier layers and TE legs [37]. To date, most researchers have aimed at improving the TE properties of Bi_2_Te_3_-based materials with little attention paid to their mechanical properties and thermal stabilities [20,35,38,39,40,41,42,43,44], both of which are critical for the module fabrication and service performance of TE modules. In this study, p-type Bi_0.5_Sb_1.5_Te_3_ zone-melted ingots were employed as the starting materials; these were then subjected to the MS-PAS process to produce densified pellets. The annealing treatment was carried out under vacuum at 473–573 K for one week to simulate and test the service performance of these materials. The temperature was set according to the possible application temperatures of TE power generation [15]. The morphologies, TE properties and mechanical responses of annealed samples were investigated and compared to those of the unannealed samples.

## 2. Results and Discussion

### 2.1. Morphologies

**Figure 1:**
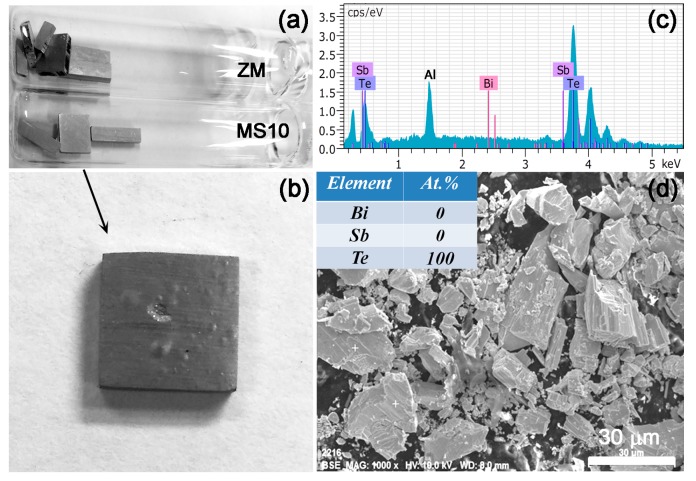
(**a**) The photograph of vacuum-sealed ZM and MS10 samples, which were annealed at 573 K for one week; (**b**) Inflations and pittings were clearly observed on the MS10 sample after annealing; (**c**,**d**) EDS results and the field-emission scanning electron microscopy (FESEM) image of the precipitates obtained from the inner wall of the quartz tube.

Figure 1a shows the general view of zone-melted (ZM) and MS10 (refer to the Materials and Methods) samples, which were vacuum-sealed in quartz tubes and then annealed at 473 or 573 K for one week. For simplicity, ZM and MS samples annealed at specific temperatures are denoted by ZM-*xx* or MS-*xx*, where *xx* stands for the annealing temperature (e.g., 473 or 573 K). The enlarged view of MS10 samples presented obvious defects (such as inflation and pitting on the surface), while ZM samples remained almost unchanged in their surface morphology. Further analysis of the densities (Table S1) can manifest these observations, given MS10 samples showed a remarkable decrease in density after annealing at 573 K; this is in contrast to the unchanged density for ZM samples before and after annealing. XRD results (Figure S1) show that annealed MS10 samples could be indexed to the Bi_0.5_Sb_1.5_Te_3_ pure phase (JCPDS#49-1713). Concurrently a small amount of precipitations were observed on the inner surface of the tube for MS10 pellets; these were confirmed to be pure elemental Te by EDS results and the field-emission scanning electron microscopy (FESEM) image in Figure 1c,d. This result was mainly ascribed to the higher saturated vapor pressure of Te compared to Bi and Sb, which led to the gradual volatilization in the annealing process [15].

**Figure 2:**
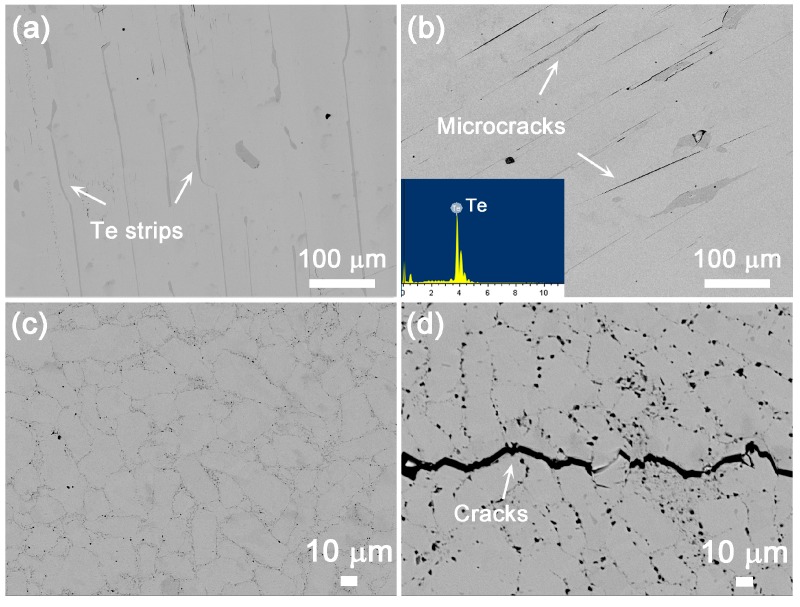
The BSE images of ZM and MS10 samples before and after annealing at 573 K: (**a**) unannealed ZM ingot; (**b**) annealed ZM ingots and MS10 samples; (**c**) before annealing; and (**d**) after annealing.

Both the ZM and MS10 pellets were characterized by back-scattered electron (BSE) imaging to analyze the phase composition and homogeneity before and after annealing (Figure 2). It is well known that overstoichiometric Te should be added in commercial p-type ZM ingots (normally Bi_2-*x*_Sb*_x_*Te_3_ + 3–8 wt % Te) to reduce the antisite defects in Sb’_Te_ and Bi’_Te_ so that the optimal hole concentration can be achieved [45,46]. The annealing process is necessary to promote the diffusion of Te atoms from precipitations into Te vacancies in the lattice. In our study, the pristine ZM materials also presented Te strips embedded in the matrix, as shown in Figure 2a. After annealing at 573 K for one week, the excess Te remained, but certain amount of microcracks initiated and extended along the interface between the lamellar structures of Bi_0.5_Sb_1.5_Te_3_ and Te strips. This can be explained by the mismatch of the coefficient of thermal expansion (CTE) between the matrix and precipitates [47,48], which resulted in the accumulation of localized thermal stresses and gradually generated microcracks [49]. The compositions from the EDS results (Table S2) confirm the phase homogeneity of ZM samples annealed at 573 K. On the other hand, it has been reported that MS-PAS is a non-equilibrium process which can generate in-situ nanostructures at the grain boundaries [21]. As depicted in the FESEM images in Figure 2 and Figure S2, the micron-sized grains of MS10 samples were decorated with nanostructured inclusions, which were presented as dark spots at grain boundaries in BSE images. These nanoprecipitations corresponded to the Bi-poor phases from the EDS results (Table S2) in accordance with our previous research [21]. Noticeably, the nanoinclusions in the MS-PAS samples grew slightly and tended to agglomerate after 573 K-annealing (Figure S2). Meanwhile, the grain boundary phase of the MS10 samples showed a remarkable decrease in the Te content, but the matrix composition remained unchanged. These results further verified the metastability of nanoprecipitates in the MS-PAS samples, and elucidated the origin of excess Te inside the quartz tube. Obvious cracks propagating along the grain boundaries were observed in annealed MS10 samples; this can also be ascribed to the CTE mismatch between the precipitates and the matrix, which likely arose from the composition fluctuation (Table S2) and random orientation of the crystals.

### 2.2. Thermoelectric Properties

Table 1 lists the room temperature electrical transport properties of the annealed ZM and MS10 samples in comparison to the unannealed samples. As is evident from Table 1, the unannealed MS10 samples presented a remarkable reduction in the hole mobility *μ*_H_ due to the enhanced carrier scattering at grain boundaries induced by the MS-PAS process, whereas the increased hole concentration of MS10 samples (in comparison with ZM samples) could be related to the slight Te evaporation during the MS and PAS processing [21]. ZM samples exhibited stable electrical performance even when annealed at 573 K for one week. For 573 K-annealed MS10 specimens, a nearly 40% increase in the Seebeck coefficient and a two-thirds decrease in electrical conductivity were presented, compared to before annealing. This was mainly ascribed to the significant reduction in the hole concentration upon annealing at 573 K. Both the mobilities for the ZM and MS10 samples remained nearly unchanged with various annealing conditions.

**Figure 3:**
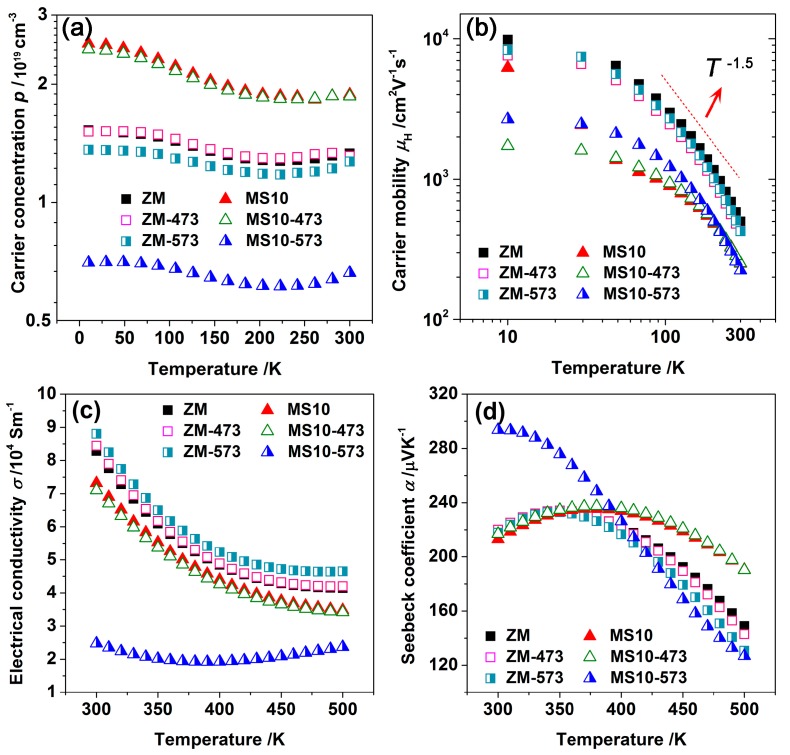
Temperature dependence of (**a**) carrier concentration; (**b**) carrier mobility; and (**c**) electrical conductivity; (**d**) Seebeck coefficient for ZM and MS10 samples before and after annealing.

The carrier concentration and mobility were measured between 10–300 K to study the carrier transport mechanisms near room temperature, as shown in Figure 3a,b. All these samples exhibited a weak temperature dependence of carrier concentration between 10 and 300 K. The significant drop in the hole concentration of the MS10-573 samples should be correlated with the Te sublimation from the non-equilibrium nanoinclusions at the grain boundaries, which likely served as donors rather than acceptors. However, further analysis of this issue is needed in order to understand the following: (a) how the holes are transferred between the matrix and nanoprecipitations; and (b) how the composition fluctuation affects the electrical properties. The conventional defects theory is insufficient to explain such a dramatic reduction in the hole concentration of MS10-573 samples because the sublimation of Te in Bi-Sb-Te alloys generally leads to enhanced antisite defects in Sb’_Te_ and Bi’_Te_, which contributes to an increased hole concentration [46]. The hole mobilities of both the ZM and MS10 samples decreased with increasing temperature and tended to follow the trend *μ*_H_ ∝ *T*^−1.5^ between 100 and 300 K, revealing the dominancy of acoustic phonon scattering. This also indicated that the annealing process did not change the mechanisms of carrier transport. MS10 pellets possessed much lower mobility than ZM ingots, likely due to the preferential crystal orientation that had been broken by the MS-PAS process; this resulted in the enhanced carrier scattering by randomly distributed crystals and increased defects concentration [21]. The electrical conductivity and Seebeck coefficient as a function of temperature are described in Figure 3c,d. MS10-573 samples showed an onset of intrinsic excitation between 350 and 400 K, and a continuous decrease in the Seebeck coefficient. This was attributed to the onset of bipolar diffusion having shifted to a lower temperature (around room temperature) due to the largely reduced hole concentration [50].

**Figure 4:**
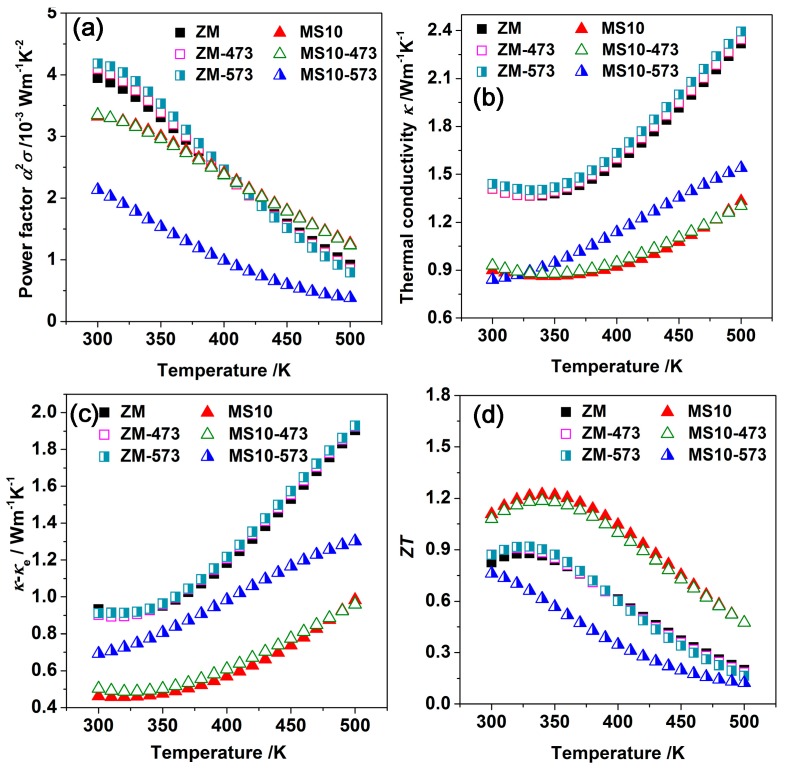
Temperature dependence of (**a**) power factor; (**b**) thermal conductivity; (**c**) lattice thermal conductivity and (**d**) *ZT* values for ZM and MS10 samples before and after annealing.

As a consequence, the power factor of the MS10-573 samples was only 2.2 × 10^−3^ W m^−1^ K^−2^ at room temperature, about 40% lower than for MS10 and MS10-473 samples (Figure 4). Due to the increased phonon scattering from in-situ nanostructures and grain boundaries, MS10 specimens exhibited much lower thermal conductivity than ZM ingots. All ZM samples followed the same trend of *K* versus *T*. In contrast to the unchanged curves for both unannealed MS10 and MS10-473 samples in thermal conductivity, their MS-573 counterparts showed a monotonic increase of *K* with rising temperature. In semiconductors with narrow bandgaps, the total thermal conductivity is given by

*K* = *K*_L_ + *K*_e_ + *K*_bi_ (1)

where *K*_L_, *K*_e_ and *K*_bi_ refer to the contribution of lattice, carrier and bipolar diffusion to the thermal conductivity, respectively [51]. Based on Wiedeman-Franz law, the electronic thermal conductivity *K*_e_ = *LσT*, where *L* is the Lorentz number. We assumed a constant *L* = 2.0 × 10^−8^ V^2^/K^2^ for degenerate semiconductors [52]. *K*_L_ and *K*_bi_ could be calculated by the subtraction of the carrier contribution *K*_e_ from the total *K*, as illustrated in Figure 4c. The *K*_L_ of the MS10 samples was only half that of the ZM ingots. Likewise, the MS10-573 specimens showed a much higher *K*_L_ than other MS10 samples, which increased monotonically with rising temperature—consistent with the variation of *K*. This was due to the low carrier concentration contributing to the low onset temperature of bipolar thermal conductivity. As a consequence, the overall *ZT* of the MS10-573 samples significantly decreased, with a maximum value of 0.75 at 300 K. In contrast, the *ZT* of MS10-473 samples was consistent with the *ZT* of unannealed samples. All the ZM samples presented similar *ZT* values regardless of the annealing temperature. Based on these results, it can be concluded that MS10 samples exhibit stable TE performance when annealed at 473 K for one week, while annealing at 573 K leads to non-negligible Te sublimation in MS10 samples, resulting in the significant degradation of TE performance.

### 2.3. Mechanical Properties

Considering that the MS10 and ZM samples exhibited excellent thermal stability when annealing at 473 K for one week, their mechanical responses (including flexural strength and compressive strength) were measured (at room temperature, 373 K, and 473 K) and compared to those of the unannealed samples. The two-parameter Weibull distribution was employed to calculate the characteristic strength and Weibull modulus; this fitted the discrete data better than the Gaussian distribution, especially for brittle materials [49]; it has been widely adopted for analyzing the mechanical properties of a series of TE materials, such as LAST-T (Ag_0.9_Pb_9_Sn_9_Sb_0.6_Te_20_) [53], skutterudites [54,55], and Bi_2_Te_3_-based alloys [20,21,43]. The original data fitted by the Weibull and Gaussion distributions are listed in Tables S3 and S4. The mechanical data with respect to the unannealed ZM and MS10 specimens can be found in our previous research [20,21]. Clearly the characteristic strengths from the Weibull distribution are slightly higher than (but comparable to) the average values obtained by the Gaussian distribution. MS10 specimens demonstrated larger Weibull moduli than ZM samples, indicating less scatter in strength values of the MS10 samples; this was mainly ascribed to the reduced manufacturing defects and enhanced machinability of the MS10 samples [21]. ZM-473 samples showed almost constant flexural strength as a function of testing temperature, while the compressive strength decreased with increasing temperature. This was mainly due to the anisotropic layer structures of the ZM samples, which led to the distinct fracture mechanisms under loading [20].

**Figure 5:**
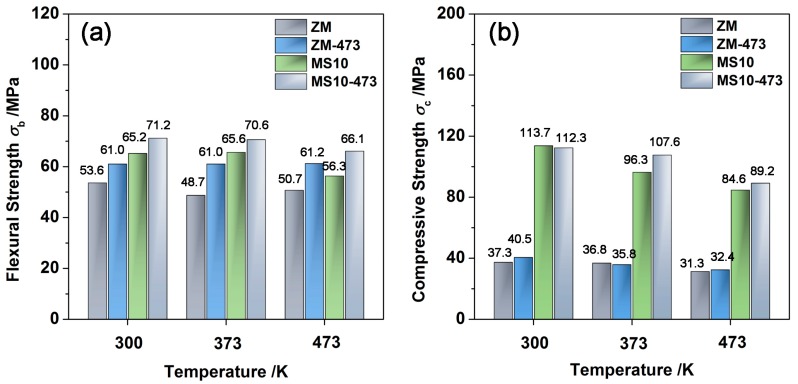
Unannealed and annealed ZM and MS10 samples as a function of testing temperature: (**a**) the bending strength and (**b**) the compressive strength.

Figure 5 describes the temperature dependence of the flexural strength and compressive strength of ZM and MS10 samples compared to the unannealed samples [21]. Ascribed to the grain refinement and hierarchical configuration by the MS-PAS process, the unannealed MS10 samples exhibited a far superior mechanical performance than for ZM samples [21]. After annealing, in comparison to their unannealed counterparts, all the ZM and MS10 samples presented more or less enhancement in their flexural and compressive strengths. This was mainly because the long-term annealing treatment gradually released the existing internal stresses inside these samples. However it is worth noting that the strength values of all these samples decreased slightly with the increase of testing temperature. This can be ascribed to the increased concentration of microcracks originating from the thermal expansion anisotropy at high temperature [21]. To summarize, this study verifies the thermal stability of p-type Bi_0.5_Sb_1.5_Te_3_ alloys prepared by the MS-PAS technique; it is expected to offer guidance for the commercial application of the alloys, and pave the way for further research into the dynamic stability to simulate the performance under vibrational stresses.

## 3. Materials and Methods

P-type Bi_0.5_Sb_1.5_Te_3_ zone melted (ZM) ingots were purchased from Thermonamic Electronics Corporation in China (Nanchang), and were then subjected to the melt spinning and plasma activated sintering (MS-PAS) process, as detailed elsewhere [21]. Melt spun samples processed with a linear speed of 10 m/s in MS and subsequent PAS were adopted as the typical objects; these exhibited superior TE and mechanical performance [21]. For brevity, this series of samples is denoted by MS10. Likewise, considering the favorable TE and mechanical properties of ZM ingots along the crystal growth direction [20], in this study, ZM samples were cut along the growth direction. The obtained MS-PAS pellets, as well as the ZM ingots, were cut into slices with specific dimensions by a linear precision saw (Isomet 4000, Buehler, Lake Bluff, IL, USA), and were polished by a grinder-polisher machine (Beta/Vector, Buehler, Lake Bluff, IL, USA). These pillars were then vacuum-sealed (~10^−2^ Pa) in quartz tubes and annealed at 473 or 573 K for one week.

The phase composition of the pellets was determined by X-ray diffraction (XRD, PRO-PANalytical Empyrean, Almelo, The Netherlands) at 40 kV and 40 mA. Morphologies of the samples were investigated by electron probe microanalysis (EPMA, JXA-8230, JEOL, Tokyo, Japan) and field-emission scanning electron microscopy (FESEM, Hitachi SU-8020, Tokyo, Japan) equipped with energy-dispersive X-ray spectroscopy (EDS, Bruker XFlash 6160, Berlin, Germany). As reported in our previous work, MS10 samples exhibit nearly isotropic TE performance, and the electrical and thermal properties were measured perpendicular and parallel to the press direction, respectively [21]. For ZM specimens, TE measurements were conducted along the crystal growth direction. The electrical conductivity (*σ*) and the Seebeck coefficient (*α*) were measured simultaneously by a standard four-probe method in the range of 300 to 500 K on the Ulvac-Riko ZEM-3 system. The low temperature Hall coefficient (*R*_H_) and low temperature electrical conductivity were characterized by a physical properties measurements system (PPMS-9, Quantum Design, San Diego, CA, USA) between 10–300 K. The carrier concentration (*n*) and the Hall mobility (*μ*_H_) were determined by equations *n* = 1/*eR*_H_ and *μ*_H_ = *σR*_H_, respectively. The total thermal conductivity was calculated from *K* = *DC*_p_*d*, where *D* is the thermal diffusivity obtained by the laser flash method (LFA-457, Netzsch, Ahlden, Germany), *C*_p_ is the specific heat measured by a differential scanning calorimeter (DSC Q20, TA Instrument, New Castle, DE, USA), and *d* is the density measured by the Archimedes’ method. DSC cycling measurement was carried out on ZM and MS10 pellets (between 300 and 723 K) with a heating/cooling rate of 10 °C/min.

The mechanical testing was performed in the same way as is illustrated in [21]. Typically, samples for mechanical testing should be polished carefully until a mirror-like finish is obtained. Both the flexural and compressive tests were conducted on a MTS universal test machine (E44.104, MTS, Shenzhen, China) with the same loading rate of 0.5 mm/min. The samples’ dimensions were fixed at 15 mm × 2 mm × 2 mm (with a fixed span of 12 mm) and 6 mm × 3 mm × 3 mm for flexural and compressive tests, respectively. The bending test was performed with the loading direction parallel to the sintering pressure for the MS10 samples, while the compressive testing was perpendicular to the press direction. With regard to the ZM specimens, the loading directions of flexural and compressive tests were perpendicular and parallel to the crystal growth direction, respectively. Around 10 prismatic bars were prepared for each test set, and the high-temperature strengths were evaluated by mounting the samples on the fixture in a furnace purged with Ar. The samples were soaked at a specific temperature (i.e., 373 K or 473 K) for 10 min before loading to retain good temperature homogeneity. Considering the wide distribution of strength statistics, the two-parameter Weibull distribution was employed to fit the data by using maximum likelihood estimations, for which 95% confidence estimates were determined [49].

## 4. Conclusions

In our study, p-type Bi_0.5_Sb_1.5_Te_3_ ZM ingots were employed as the starting materials; these were then subjected to the MS-PAS technique to obtain the densified pellets. The thermal stability of these samples was investigated by vaccum-annealing at 473 K and 573 K for one week. The results show that the ZM samples exhibited excellent stability in TE and mechanical properties, which were almost independent of annealing temperature. In contrast, MS-PAS samples demonstrated a considerable decrease in the TE performance when annealed at 573 K for one week. The 473 K-annealed MS-PAS samples displayed nearly unchanged TE properties and even a slight increase in the mechanical responses, in comparison to their unannealed counterparts. The current work has paved the way for the reliable applications of p-type Bi_0.5_Sb_1.5_Te_3_ specimens prepared by the MS-PAS technique.

## Figures and Tables

**Table 1:** The room temperature electrical transport properties of ZM and MS10 samples before and after annealing at 473 and 573 K, respectively.

Samples	Annealing Temperature	*α μ*VK^−1^	*σ* 10^4^ S m^−1^	*p* 10^19^ cm^−3^	*μ*_H_ cm^2^ V^−1^ s^−1^
ZM	Unannealed	218	8.3	1.3	390
	473 K	220	8.4	1.3	404
	573 K	218	8.8	1.3	423
MS10	Unannealed	213	7.3	1.9	242
	473 K	217	7.1	1.9	238
	573 K	294	2.5	0.7	234

## Supplementary Materials

The following resources are available online at www.mdpi.com/1996-1944/10/6/617/s1. Figure S1: The XRD patterns of MS10 samples before and after annealing at 473 K and 573 K; Figure S2: The FESEM images of MS10 samples before and after annealing at 573 K; Table S1: The densities of ZM and MS10 samples before and after annealing at 473 K and 573 K; Table S2: The composition of ZM and MS10 samples before and after annealing at 573 K; Table S3: The table lists the number of samples measured at room temperature, 373 K and 473 K, and the temperature dependent bending strength of annealed ZM and MS-PAS samples fitted by Weibull and Gaussian distributions; Table S4: The table lists the number of samples measured at room temperature, 373 K and 473 K, and the temperature dependent compressive strength of annealed ZM and MS-PAS samples fitted by Weibull and Gaussian distributions.
