# Supplementary material for: Thermal Stability of P-Type BiSbTe Alloys Prepared by Melt Spinning and Rapid Sintering
Source: Materials (Basel). 2017 Jun 6;10(6):617. doi: 10.3390/ma10060617 (PMC5553525; doi:10.3390/ma10060617)
Supplement: Supplementary file 1 [file materials-10-00617-s001.pdf]

Supplementary Materials: Thermal stability of p-type BiSbTe alloys prepared by melt spinning and rapid sintering

Yun Zheng, Gangjian Tan, Yubo Luo, Xianli Su, Yonggao Yan, and Xinfeng Tang

Table S1. The densities of ZM and MS10 samples before and after annealing at 473 K and 573 K.

Samples	Density /gcm^{-3}		
	Unannealed	473 K annealed	573 K annealed
ZM	6.80	6.81	6.81
MS10	6.81	6.82	6.58

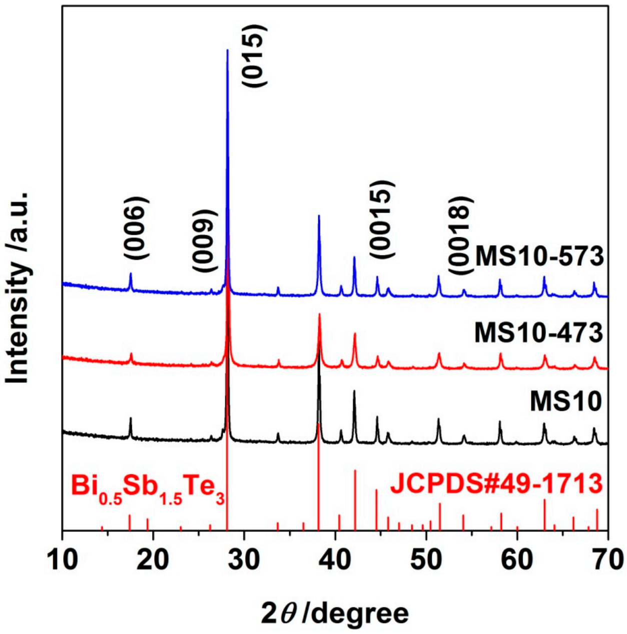

Figure S1. The XRD patterns of MS10 samples before and after annealing at 473K and 573K.

Table S2. The composition of ZM and MS10 samples before and after annealing at 573 K.

Samples	Composition by EDS	
	Unannealed	573 K annealed
ZM matrix	$Bi_{10.2}Sb_{29.8}Te_{60.0}$	$Bi_{9.5}Sb_{29.1}Te_{61.4}$
MS10 matrix	$Bi_{10.1}Sb_{29.5}Te_{60.3}$	$Bi_{10.1}Sb_{29.6}Te_{60.3}$
MS10 precipitates	$Bi_{6.8}Sb_{23.1}Te_{70.1}$	$Bi_{8.5}Sb_{39.1}Te_{52.6}$

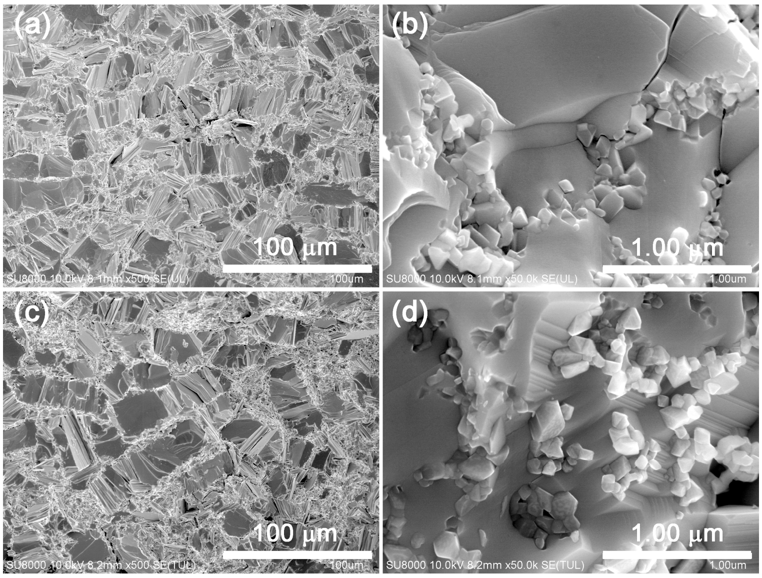

Figure S2. The FESEM images of MS10 samples: (a) and (b) before annealing at 573K, and (c) and (d) after annealing at 573K.

Table S3. The table lists the number of samples measured at room temperature, 373 K and 473 K, and the temperature dependent bending strength of annealed ZM and MS-PAS samples fitted by Weibull and Gaussian distributions.

Sample	*T*/K	Number	Weibull distribution		Gaussian distribution	
			Characteristic strength /MPa	Weibull modulus	Ave. /MPa	Std dev.
ZM	300	10	61.0 (34.3, 69.9)	8.5 (6.4, 10.7)	56.7	9.7
	373	10	61.0 (46.1, 69.8)	6.7 (3.6, 9.8)	57.5	7.7
	473	10	61.2 (44.9, 67.4)	6.0 (4.4, 7.6)	57.4	8.5
MS10	300	11	71.2 (54.9, 78.5)	9.9 (8.3, 11.6)	68.0	7.2
	373	9	70.6 (58.8, 76.9)	11.4 (9.8, 13.1)	68.0	5.9
	473	9	66.1 (51.5, 73.7)	7.9 (6.5, 9.4)	62.8	7.6

Table S4. The table lists the number of samples measured at room temperature, 373 K and 473 K, and the temperature dependent compressive strength of annealed ZM and MS-PAS samples fitted by Weibull and Gaussian distributions.

			Weibull distribution		Gaussian distribution	
**Sample**	***T*/K**	**Number**	**Characteristic strength /MPa**	**Weibull modulus**	**Ave. /MPa**	**Std dev.**
ZM	300	11	40.5 (22.1, 68.0)	2.5 (2.1, 2.9)	36.5	14.1
	373	10	35.8 (22.8, 45.8)	5.2 (3.1, 7.3)	33.1	6.5
	473	10	32.4 (19.8, 43.1)	3.4 (2.2, 4.6)	29.5	8.1
MS10	300	10	112.3 (94.2, 119.0)	10.2 (7.5, 13.0)	108.0	9.6
	373	10	107.6 (84.8, 115.9)	12.2 (10.7, 13.7)	103.3	9.5
	473	10	89.2 (65.3, 105.6)	5.9 (5.1, 6.8)	83.8	13.2
